# Mutant and non-mutant neoantigen-based cancer vaccines: recent advances and future promises

**DOI:** 10.37349/etat.2022.00111

**Published:** 2022-12-22

**Authors:** Mohamad Omar Ashi, Fathia Mami-Chouaib, Stéphanie Corgnac

**Affiliations:** INSERM UMR 1186, Integrative Tumor Immunology and Immunotherapy, Gustave Roussy, Fac. de Médecine - Univ. Paris-Sud, Université Paris-Saclay, 94805 Villejuif, France; University of Salford, UK

**Keywords:** Cancer immunotherapy, therapeutic peptide vaccine, messenger RNA vaccine, tumor-associated antigen, neoantigen, T-cell epitopes associated with impaired peptide processing

## Abstract

Major advances in cancer treatment have emerged with the introduction of immunotherapies using blocking antibodies that target T-cell inhibitory receptors, such as programmed death-1 (PD-1) and cytotoxic T-lymphocyte-associated antigen-4 (CTLA-4), known as immune checkpoints. However, most cancer patients do not respond to immune checkpoint blockade (ICB) therapies, suggesting the development of resistance mechanisms associated with either an insufficient number of preexisting tumor-specific T-cell precursors and/or inappropriate T-cell reactivation. To broaden clinical benefit, anti-PD-1/PD-1 ligand (PD-L1) neutralizing antibodies have been combined with therapeutic cancer vaccines based on non-mutant and/or mutant tumor antigens, to stimulate and expand tumor-specific T lymphocytes. Although these combination treatments achieve the expected goal in some patients, relapse linked to alterations in antigen presentation machinery (APM) of cancer cells often occurs leading to tumor escape from CD8 T-cell immunity. Remarkably, an alternative antigenic peptide repertoire, referred to as T-cell epitopes associated with impaired peptide processing (TEIPP), arises on these malignant cells with altered APM. TEIPP are derived from ubiquitous non-mutant self-proteins and represent a unique resource to target immune-edited tumors that have acquired resistance to cytotoxic T lymphocytes (CTLs) related to defects in transporter associated with antigen processing (TAP) and possibly also to ICB. The present review discusses tumor-associated antigens (TAAs) and mutant neoantigens and their use as targets in peptide- and RNA-based therapeutic cancer vaccines. Finally, this paper highlights TEIPP as a promising immunogenic non-mutant neoantigen candidates for active cancer immunotherapy and combination with TAA and mutant neoantigens. Combining these polyepitope cancer vaccines with ICB would broaden T-cell specificity and reinvigorate exhausted antitumor CTL, resulting in the eradication of all types of neoplastic cells, including immune-escaped subtypes.

## Introduction

Current cancer immunotherapies are designed to boost spontaneous antitumor T-cell response either via 1) administration of blocking monoclonal antibodies targeting T-cell inhibitory receptors, such as cytotoxic T-lymphocyte-associated antigen-4 (CTLA-4) and programmed death-1 (PD-1) [1, 2]; 2) adoptive cell transfer of *in vitro* expanded native tumor-specific T cells or engineered T lymphocytes transformed to express chimeric antigen receptors (CARs) or T-cell receptor (TCR) targeting malignant cells [3, 4]; or 3) therapeutic vaccination of cancer patients with shared tumor-associated antigens (TAAs) or mutant antigens [5–7]. In the latter setting, for the design of therapeutic cancer vaccines, TAA recognized by tumor-specific cytotoxic T lymphocytes (CTLs), isolated either from the patient’s tumor or peripheral blood lymphocytes (PBL), have been identified using genetic and biochemical approaches [5]. More recently, accessibility to next-generation sequencing (NGS) technology and *in silico* epitope prediction algorithms, has permitted the identification of tumor neoantigens arising from gene mutations that are expressed exclusively by malignant cells. These mutant neoantigens have opened up new perspectives in active immunotherapy to a wide range of cancer types without the need for isolating tumor-reactive CTL clones and establishing autologous cancer cell lines [8, 9].

It is generally agreed that CTL is major effector of adaptive T-cell immunity and an ideal weapon to specifically combat cancers. They are capable of destroying transformed cells upon recognition, via the TCR, of specific epitopes presented on the target surface by major histocompatibility complex class I (MHC-I)-beta-2-microglobulin (β_2_m) complexes. A CTL response to tumor cells was demonstrated by isolating CD8^+^ T cells from patients with cancers such as melanoma and lung carcinoma, capable of mediating specific cytotoxic activity against autologous tumor cells [10, 11]. Detection of TAA-reactive CD8^+^ T lymphocytes in spontaneously regressing melanomas further strengthened the concept of tumor-specific CTL immunity [10]. Remarkably, high tumor infiltration by CD8^+^ T cells and CD8^+^CD103^+^ resident memory T cells correlated with better survival for treatment-naive cancer patients and, to some extent, improved response to immune checkpoint blockade (ICB) [12–14]. Response to ICB immunotherapy has been associated with the presence of T cells directed against mutant neoantigens [15–17]. These neoantigens are highly immunogenic because they are only expressed by transformed cells and thus bypass central thymic tolerance [18]. The present review summarizes advances in the field of mutant and non-mutant tumor neoantigen identification for their use in active immunotherapy and highlight new trends in therapeutic cancer vaccines based on shared non-mutant neoepitopes, known as T-cell epitopes associated with impaired peptide processing (TEIPP) [19, 20].

## TAA-based therapeutic cancer vaccines: disappointments and promises

**Figure 1.**
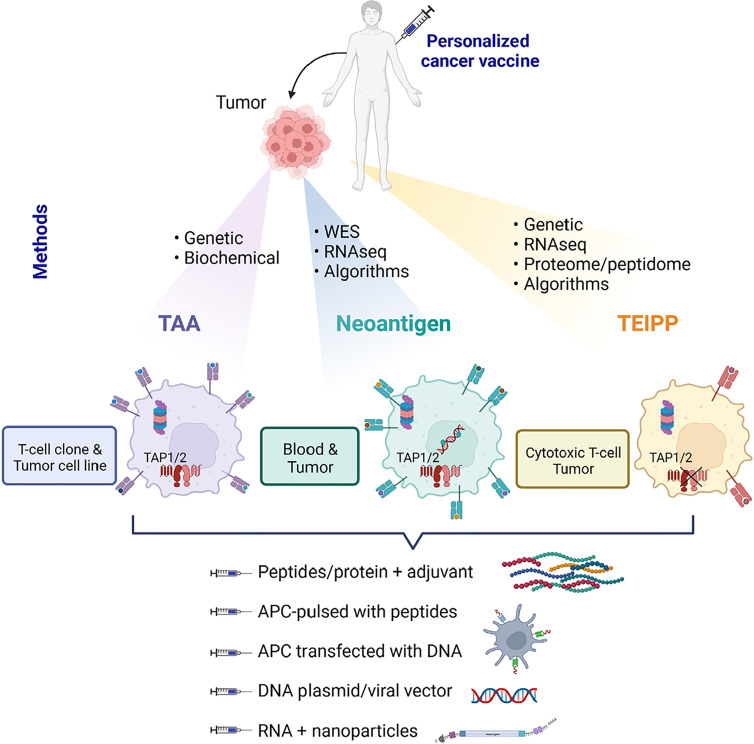
Therapeutic cancer vaccines based on shared TAA, mutant neoantigens, and non-mutant neoantigens, named TEIPP. Methods of identification and required biological materials are described and specified for each type of antigen. RNAseq: RNA sequencing; TAP1/2: transporter associated with antigen processing 1 and 2; APC: antigen-presenting cell; WES: whole exome sequencing

The identification in the early 1990s of TAA recognized by T lymphocytes represents a paramount advance in our knowledge of cancer immune surveillance and the antitumor T-cell response. It also opened up new perspectives in the field of immunooncology and cancer immunotherapy. The first human tumor antigen recognized by autologous CTL, named melanoma-associated antigen-1 (MAGE-1), was identified using a genetic method [21]. Subsequently, several additional TAA recognized by tumor-reactive T cells were cloned [5]. According to their pattern of expression, tumor antigens were classified into at least five groups: 1) cancer-germline antigens, including MAGE-1, which are expressed by tumors as well as adult reproductive tissues; 2) differentiation antigens, such as melanosomal differentiation antigens that are shared between melanoma and melanocytes, and refer to antigens detected at particular phases of cell differentiation; 3) overexpressed antigens, normal proteins that are expressed at much higher levels in tumors than in healthy tissues; 4) mutant antigens, also known as neoantigens, arising from somatic mutations and thus expressed exclusively by cancer cells; and 5) viral antigens, derived from viral proteins that are the origin of several types of cancers, including cervical carcinoma, nasopharyngeal carcinoma, and hepatocarcinoma (Table 1 and Figure 1) [5, 22].

**Table 1.** Classification of cancer antigens

**Type of antigens**	**Antigen characteristics**	**Example of human tumor antigens**
Cancer-germline	Expressed by tumor cells and adult reproductive tissues	MAGE, BAGE, NY-ESO-1
Differentiation	Expressed by tumors cells and a limited range of normal tissues	Tyrosinase, Melan-A/MART-1, Gp100, CEA
Overexpressed	Highly expressed in tumor cells and few in some normal tissues	EGFR, HER2, MUC1
Mutant neoantigens	Mutant antigens, expressed only by tumor cells as a result of mutation, patient-specific	p53, Ras, BCR-ABL, ACTN4
TEIPP neoantigens	Non-mutated antigens expressed by tumor cells with APM defects	ppCT, LRPAP1
Viral antigens	Expressed only by tumor cells as a result of viral infection	HPV E6–E7, EBV, HBV, HTVL

EGFR: epidermal growth factor receptor; HER2: human EGFR 2; ppCT: preprocalcitonin; HPV: human papillomavirus; EBV: Epstein-Barr virus; HBV: hepatitis B virus; HTVL: human T cell leukemia virus; APM: antigen presentation machinery; BAGE: B melanoma antigen; NY-ESO-1: New York esophageal squamous cell carcinoma 1; Gp100: glycoprotein 100; CEA: carcinoembryonic antigen; MUC1: mucin 1; p53: tumor protein 53; BCR-ABL: breakpoint cluster region and abelson oncogene; LRPAP1: LDL-receptor-related protein-associated protein 1; ACTN4: actinin 4

TAA was used for the development of therapeutic cancer vaccines aimed at priming and/or strengthening a preexisting antitumor CTL response [23]. Unlike prophylactic cancer vaccines that are designed to prevent virus-induced cancers, such as HPV-induced cervical cancers [24], the design of therapeutic cancer vaccines has proved to be much more challenging. TAA-based vaccines face the problem of immune tolerance to self-antigens and suppression induced by the tumor itself or immunosuppressive cells [25]. In this context, several DNA, RNA, and synthetic peptide vaccines have been produced to stimulate the immune system against TAA, including cancer-testis antigens, such as MAGE-A1, differentiation antigens, including tyrosinase-related protein-2 (TRP-2) and Melan-A/MART-1, and viral antigens, such as HPV [26]. Tumor antigens used in the form of peptides or recombinant proteins, delivered with a potent adjuvant, were able to elicit efficient antitumor T-cell responses [27]. In addition, TAA could be expressed in non-malignant cells, meaning that the risk of vaccine-induced autoimmunities, such as vitiligo, could occur.

The efficacy of peptide-based vaccines is dependent on the vaccination route, the quality of the adjuvant used, and the length of the synthetic peptides. While short synthetic peptides (8–11 amino acids) bind directly to human leukocyte antigen class I (HLA-I) molecules to prime antigen-specific CD8^+^ T-cells, long peptides (25–50 amino acids) must be processed by APCs to trigger a specific T-cell response [28, 29]. In contrast to short peptides, vaccine formulations with long peptides induce both CD8^+^ and CD4^+^ T-cell immunity, leading to a stronger antitumor response [26, 30–32]. Moreover, multi-epitope vaccination promotes CD4^+^ T-cells and helps to generate potent CTL and broaden the CD8^+^ T-cell repertoire. Furthermore, the development of messenger RNA (mRNA)-based vaccines offer promising opportunities in cancer therapeutics. In this regard, technological innovations have made mRNA an attractive tool candidate with rapid, inexpensive, and large-scale production compared to other vaccines [33]. Although mRNA could be sensitive to degradation and not internalized by dendritic cells (DCs), several efforts have been made to produce stable mRNA and reduce the non-specific activation of innate immunity due to residual double-strand contaminations [34]. Indeed, mRNA is degraded by normal cellular processes, and their *in vivo* half-life can be regulated by various delivery methods and modifications [35]. To this aim, synthetic DNA fragments encoding putative non-mutant neoepitopes connected by non-immunogenic glycine/serine linkers are cloned into a starting vector, and then the DNA is linearized and subjected to *in vitro* transcription [36, 37]. The adjuvant added in the vaccine formulation will also determine the efficacy of the vaccine by promoting the maturation of DC and thereby optimizing antigen delivery to T cells and their subsequent activation. To date, the activation of DC via targeting of toll-like receptors (TLRs) with cytosine-phosphate-guanine (CpG) motifs, lypopeptides, or mRNA demonstrated potent results [38]. Another concern for the vaccine design is to protect tumor antigens from biodegradation during vaccine delivery. For a long period, alum adjuvant which creates a depot at the injection site was used to protect antigens and enable their prolonged exposure to activate innate cell immunity. Nanoparticles are now used as vaccine carriers, resulting in more effective vaccine delivery and antigen uptake by DC [39]. Particularly used in the context of mRNA vaccines, nanotechnologies have led to innovative and faster vaccine development that demonstrated a high efficiency [39–42]. Recently, a novel broad-spectrum neoantigen vaccine delivery system based on β-1,3-glucan particles and derived from natural edible *Saccharomyces cerevisiae* showed strong activation of immune cells that inhibited tumor growth in various syngeneic mouse models [43].

First-generation peptide vaccines with non-mutant TAA, such as MAGE-A3, NY-ESO-1, tyrosinase, TRP-2, or MART-1 delivered with an adjuvant or pulsed on autologous or allogeneic DC, resulted in clinical responses in only a limited number of cancer patients [23, 44–46]. For instance, a MAGE-A3-based vaccine developed in patients with lung cancer reduced the risk of relapse but did not increase disease-free survival compared with a placebo [47]. Another therapeutic vaccination conducted with the preferentially expressed antigen in melanoma (PRAME) antigen did not result in objective cancer regression or an increase in disease-free survival in patients with non-small cell lung cancer (NSCLC) [48]. Peptide vaccines targeting the MUC1 antigen did not enhance overall survival compared to placebo, although they improved median survival as well as the effect of chemotherapy, which correlated with induction of CTL responses to targeted and non-targeted TAA [49–52]. In addition, multi-peptide vaccines for patients with advanced NSCLC did not improve survival or showed only a minimal benefit for overall survival (Table 2) [53, 54]. More recently, RNA vaccines, which target four non-mutant TAA, combined with ICB, demonstrated clinical responses some of which were accompanied by the induction of a strong CD4 and CD8 T-cell immunity to the vaccine antigens [40]. However, despite encouraging results in initial clinical trials with TAA-based cancer vaccines, with activation of a specific CTL response, most phase 3 trials have not observed the expected results in terms of survival benefits, in particular in late-stage patients with treatment-refractory tumors [6, 55]. Thus, second-generation cancer vaccines, based on tumor mutant neoantigens that are selectively presented by cancer cells, have been designed for the treatment of a wide range of cancer types with promising results expected.

**Table 2.** Clinical trials with TAA and neoantigens-based cancer vaccines encoding

**Organ**	**Cancer type/stage**	**Phases**	**Cancer vaccine**	**TAA/neoantigens**	**Formulation**	**Study results**	**Sponsor**	**NCT number**	**Reference**
Lung	NSCLC Stage IIIB or IV	Phase IIB trial	TG4010	MUC1	TG4010: recombinant modified vaccinia virus strain Ankara (MVA) encoding MUC1 and human interleukin 2 (IL-2)	TG4010 enhances the effect of chemotherapy in advanced NSCLC patients	Transgene SA (France)	NCT00415818	[50]
Lung	NSCLC Stage IIIA *vs.* IIIB	START study: phase III trial	Stimuvax	MUC1	Tecemotide: MUC1-derived 25-amino acid BLP25 lipopeptide, immunoadjuvant monophosphoryl lipid A, and three liposome-forming lipids	No significant difference in overall survival for all patients	Merck KgaA (Germany)	NCT00409188	[49]
Lung	NSCLC Stage IB, II and IIIA	Phase III trial	MAGE-A3 (AS15 and AS02B)	MAGE-A3	Adjuvant treatment with MAGE-A3	MAGE-A3 immunotherapeutic use in NSCLC has been stopped	GlaxoSmithKline (GSK) Biologicals SA (UK)	NCT00480025	[47]
Lung	NSCLC Stage IB to IIIA	Phase I dose escalation	PRAME (with AS15)	PRAME	PRAME recombinant protein with AS15	Anti-PRAME humoral responses with no cancer regression stopped due to negative results	GSK Biologicals SA (UK)	NCT01159964	[48]
Lung	NSCLC Stage IIIB/IV	Phase II trial	Personalized peptide vaccine with docetaxel	Several TAA	31 Peptides from several TAA	Positive predictive value (PPV) may be efficacious for the humoral immunological responders but did not improve survival in combination with docetaxel for NSCLC patients	Kurume University Research Center for Innovative Cancer Therapy Kurume University School of Medicine (Japan)	UMIN Clinical Trials Registry (UMIN number 000003521)	[53]
Skin	Melanoma Stage IIIA-C/IV	Phase I trial	IVAC mutanome	Multiple neoantigens	Poly-neo-epitopic coding RNA vaccine	Immune responses to the majority of neoantigens contained in the vaccine/vaccine-induced T-cell responses in all vaccinated melanoma patients	BioNTech RNA Pharmaceuticals GmbH (Germany)	NCT02035956	[37]
Lung	NSCLC Stage IV	TIME study: phase IIB/III trial	TG4010	MUC1	TG4010: recombinant MVA encoding MUC1 and human IL-2	TG4010 modulates CD8^+^ T-cell response with improvements in clinical outcome	Not applicable	NCT01383148	[52]
Brain	Glioblastoma Stage IV	Phase I trial	Actively personalized vaccine 1 (APVAC1)	7 Non-mutated peptides, 1 viral peptide, and 2 tumor-associated peptides	APVAC1 vaccine plus poly-ICLC (Hiltonol^®^) and GM-CSF	Unmutated APVAC1 antigens elicited sustained responses of central memory CD8^+^ T cells	Immatics Biotechnologies GmbH (Germany)	NCT02149225	[56]
Brain	Glioblastoma Stage IV	Phase I trial	Glioblastoma personalized peptide vaccine (GBM PVax)	8 Synthetic long peptides targeting seven neoantigens	Personalized neoantigen-based long peptide vaccine with poly-ICLC (Hiltonol^®^)	Specific T-cell responses in the blood	Washington University School of Medicine (USA)	NCT02510950	[57]
Brain	Glioblastoma Stage IV	Phase I/IB trial	Personalized neoantigen targeting vaccine	20 Long peptides divided into pools of 3–5 peptides admixed with poly-ICLC	Neoantigen vaccine with radiation therapy plus pembrolizumab	Polyfunctional neoantigen-specific CD4^+^ and CD8^+^ T-cell responses exhibiting memory phenotype	Dana-Farber Cancer Institute (USA)	NCT02287428	[58]
Lung	NSCLC Stage IV	Phase II trial	Tedopi^®^	5 TAA (CEA, p53, HER2/neu, MAGE2 and MAGE3)	Tedopi plus docetaxel or tedopi plus nivolumab	T-cell response with a better survival rate	OSE Immunotherapeutics (France)	NCT04884282	[54]
Digestive system	Gastrointestinal Stage IV	Phase I/II trial	mRNA-4650	Up to 20 different neoantigens	Neoantigen mRNA-based cancer vaccine	mRNA-4650 vaccine was safe and elicited mutation-specific T-cell responses against predicted neoepitopes	National Cancer Institute (USA)	NCT03480152	[59]
Skin	Melanoma Stage IIIB, C, and IV	Phase I (Lipo-MERIT trial)	FixVac (BNT 111)	4 TAA (NY-ESO-1, MAGE-A3, tyrosinase, and TPTE)	Liposomal RNA (RNA-LPX) vaccine	Strong CD4^+^ and CD8^+^ T-cell immunity against the vaccine antigens/antigen-specific cytotoxic T-cell responses in some responders reach magnitudes and are durable	BioNTech SE (Germany)	NCT02410733	[40]
Lung	Advanced lung cancer Stage IV	Phase I trial	Neo-DCVac	13–30 Peptide-based neoantigens	Neoantigen-pulsed DC vaccine	Neo-DCVac was well tolerated, safe, and capable of eliciting specific T-cell immunity and therapeutic benefit	Sichuan University (China)	NCT02956551 and Chinese Clinical Trial Registry (ChiCTRONC-16009100)	[60]
Skin	Melanoma Stage IIIB/C and IVM1a/b	Phase I trial	NeoVax	Long-peptide vaccine targeting up to 20 personal neoantigens	Neoantigen vaccine with poly-ICLC (Hiltonol^®^)	T-cell responses with *ex vivo* detection of neoantigen-specific T cells exhibiting memory phenotype	Patrick Ott, MD (USA)	NCT01970358	[61]
Brain	Glioma Stage III/IV	Phase I trial	Isocitrate dehydrogenase type 1 (IDH1)-vac	20-Mer peptide-based neoantigens encompassing IDH1R132H-mutated region	IDH1 peptide vaccine with Montanide^®^	IDH1 was immunogenic and induces specific T helper cell responses	National Center for Tumor Diseases (Germany)	NCT02454634	[62]
Pancreas	Pancreatic carcinoma Stage IV	Phase I trial	iNeo-Vac-P01	5–20 Peptides-based neoantigens	Neoantigen-based peptide vaccine (iNeo-Vac-P01) with adjuvant (GM-CSF)	No severe vaccine-related adverse effects/higher peripheral IFN-γ titer and CD4^+^ or CD8^+^ effector memory T cells count post-vaccination	Zhejiang Provincial People’s Hospital (China)	NCT03645148	[63]

GM-CSF: granulocyte-macrophage colony-stimulating factor; IFN-γ: interferon-γ

## Neoantigens as promising targets for cancer immunotherapy

WES combined with RNAseq and T-cell epitope prediction programs permitted the identification of cancer-specific antigens generated by somatic mutations in individual patient tumors [64]. These neoantigens have been fundamental to the success of multiple immunotherapy strategies, including ICB [15–17], adoptive cell transfer [65–67], and DC-based cancer vaccines [68, 69], and are considered predictive biomarkers of clinical response to therapies. Thus, two immunodominant neoantigens have been identified in methylcholanthrene (MCA)-induced sarcoma model, and mutant tumor antigen-specific T cells showed to be reactivated upon immunotherapy with PD-1 and/or CTLA-4 blockade, enabling an effective tumor rejection that could be boosted with neoepitope-based vaccination [70]. Comparison of neoantigen-pulsed DC vaccines with the neoantigen-adjuvant vaccine in mouse tumor models demonstrated that, while 4/6 of neoantigen-adjuvant vaccines induced significant neoantigen-specific CD8^+^ T-cell response, 6/6 of neoantigen-pulsed DC-based vaccines induced strong T-cell response [71].

In humans, mutant neoantigens were used for the design of personalized immunotherapy aimed at specifically targeting tumors of individual patients [64]. It should be noted that the selection of neoantigens for the candidate vaccine is an important step, as not all mutant protein sequences are adequately processed by cancer cells and are potent activators of T lymphocytes. Moreover, the quality of a neoantigen, its similarity with self-antigens, and the type of mutation and HLA restriction element could impact the ability of a given antigen to elicit a T-cell response and thus ICB outcome [72–76]. Remarkably, therapeutic cancer vaccines generated with mutant neoantigens observed clinical benefits in some cancers, such as melanoma [36, 37] and lung cancer [77, 78], and resulted in increased clonal diversity of neoantigen-specific T lymphocytes [69]. Clinical trials performed in glioblastoma [56–58] and advanced solid tumors [79] demonstrated the safety and feasibility of personalized vaccines with the capacity of initiating tumor-specific T-cell responses. Moreover, personalized mRNA-based vaccines against gastrointestinal cancer elicited both CD8 and CD4 neoantigen-specific T-cell responses to the predicted neoepitopes [59]. Another personalized neoantigen long-peptide vaccine led to the expansion of neoepitope-specific CD8 and CD4 T lymphocytes in the primary tumor and metastases of an NSCLC patient with low tumor mutational burden (TMB) (Table 2) [80].

Unfortunately, most personalized peptide vaccines do not improve the survival of patients with advanced NSCLC [53]. The limited success of neoantigen-based vaccination approaches may be due to the suboptimal reactivation of tumor neoepitope-specific T cells in an immunosuppressive tumor microenvironment [81]. It may also be associated with preexisting and acquired resistance mechanisms that impede T-cell functions and lead to tumor escape from the immune system [82, 83]. To bypass resistance mechanisms, neoantigen-based vaccination was combined with the depletion of regulatory T cells (Tregs) in murine cancer models [84]. In this context, a combination of an IL-2 pathway agonist with Treg-depleting activity with the cancer vaccine led to the activation of neoantigen-specific T cells and a complete eradication of the tumor [84]. With regard to the issue of the low frequency of neoantigen-specific T cells, engineered IL-2 receptor (IL-2R) agonists have been designed to expand T lymphocytes and take advantage of IL-2 benefits [85]. Expression of inhibitory receptors on activated tumor-specific T lymphocytes and their ligands on tumor cells is also a major constraint resulting in cancer immune evasion [86, 87]. To reverse the exhausted state of vaccine-induced T lymphocytes and to promote T-cell proliferation and reactivation, therapeutic vaccines were combined with ICB. Immunization of melanoma patients with personalized neoepitope peptide vaccines or RNA vaccines combined with PD-1 blockade resulted in tumor regression with amplification of neoantigen-specific T-cell repertoires [36, 37]. Another personalized vaccine combined with anti-PD-1 was evaluated in metastatic lung cancer with neoantigen peptide-pulsed autologous DC, resulting in objective responses in some patients, which correlated with the induction of neoantigen-specific T-cell responses [60]. Recently, the adoptive transfer of neoantigen-reactive T-cells has demonstrated promising results in mouse cancer models. In this regard, *in vivo* transfer of mutation-specific T cells induced by RNA mutanome vaccine resulted in beneficial antitumor effects [88]. In hepatocellular carcinoma (HCC) patients, a prospective clinical trial exploring a new combinatorial approach with a neoantigen-loaded DC vaccine and neoantigen-activated T-cell therapy used as an adjuvant demonstrated good feasibility and low toxicity, with neoantigen-specific responses [89]. However, relapse, such as that due to the outgrowth of β_2_m-deficient malignant cells as an acquired resistance mechanism, was observed in responding melanoma patients [40]. Indeed, the downregulation of APM components, including the loss of MHC-I molecules and β_2_m, is frequently used by tumors to evade CTL recognition and elimination. Among additional mechanisms used by malignant cells to escape specific CD8 T lymphocytes, alterations in TAP play an important role by inducing a severe decrease in the expression of MHC-I/β_2_m-peptide complexes on the surface of cancer cells enabling escape from TCR-mediated cytotoxicity [90–94]. Consequently, the design of innovative cancer vaccines based on non-mutant neoepitopes that are specifically processed by tumor cells carrying defects in APM opens new perspectives in active immunotherapy [19, 20].

## TEIPP: promising targets for immune escaped tumors

Most antigenic peptides recognized by CTL originate from the degradation of intracellular proteins in proteasomes, and the transport of the generated peptides from the cytosol into the endoplasmic reticulum by TAP complexes. Peptides of nine to ten amino acids then bind to MHC-I-β_2_m complexes and are presented on the membrane of APCs for CD8 T-cell recognition. However, under continuous CTL pressure, cancer cells frequently downregulate TAP1 and/or TAP2 to evade T-cell destruction [95, 96]. Indeed, defects in TAP subunits result in a sharp decrease in surface expression of MHC-I/peptide complexes, thereby creating an ‘invisible’ tumor phenotype enabling resistance of MHC-I^low^ cancer cells to TCR-mediated killing. Remarkably, an alternative antigenic peptide repertoire, referred to as TEIPP, arises on these MHC-I^low^ neoplastic cells [19]. These epitopes are processed independently of the proteasome/TAP pathway, representing a precious reserve allowing selective elimination of immune-edited tumors that have acquired resistance to specific CD8 T lymphocytes (Figure 1). They are derived from ubiquitous non-mutant self-proteins that are not naturally loaded into MHC-I molecules of healthy cells because they express standard levels of TAP [97]. In this context, we previously identified a tumor antigen, the ppCT, which includes the first known human TEIPP (ppCT_16–25_) [95, 98]. The ppCT_16–25_ epitope was recognized on the HLA-A2^low^ NSCLC cell line by an autologous CTL clone isolated from patient tumor-infiltrating lymphocytes. It is derived from the C-terminal region of the leader sequence of the ppCT precursor protein and is processed independently of TAP, by signal peptidase and signal peptide peptidase [95, 98]. This antigen-processing pathway represents an alternative mechanism exploited by the immune system to eliminate TAP-deficient tumor variants. More recently, additional human non-mutant neoantigens presented by TAP-deficient tumors were identified using a combinatorial screening approach and algorithm-based predictions [97]. These non-mutant neoepitopes, together with the ppCT_16–25_, represent attractive candidates for therapeutic vaccines targeting immune-escape tumors. Indeed, tumors often evade CD8 T-cell immunity by downregulating TAP1 and/or TAP2 peptide transporters. In this context, TEIPP constitutes a category of immunogenic non-mutant neoantigens that emerge during tumor immune evasion, and thus correspond to promising candidates for cancer immunotherapy [99, 100].

The development of innovative immunotherapies based on tumor non-mutant neoantigens that are selectively presented by malignant cells carrying APM defects and that are competent in inducing a specific CTL response able to destroy such transformed cells represents both a major challenge and a new hope in cancer treatment. In this context, we recently provided proof of concept of a ppCT peptide vaccine based on a cocktail of five immunogenic peptides, including the ppCT_16–25_ TEIPP, delivered with poly(I:C) adjuvant. This active immunotherapy was able to induce efficient antitumor T-cell responses against APM-impaired tumors transplanted into HLA-A*0201/HLA-DR3-transgenic (HHD-DR3) mice and NOD-*scid*-*Il2rγ*^null^ (NSG) mice adoptively transferred with human HLA-A2^+^ peripheral blood mononuclear cells (PBMCs). This resulted in the control of tumor growth and regression of established tumors expressing low levels of HLA-A2/ppCT complexes [100]. Along the same lines, a TAP-independent signal peptide was able to induce CD8 T-cell immunity in escaped tumors upon anchor replacement, leading to efficient cross-presentation [101]. These results support the conclusion that signal sequence-derived peptides are attractive candidates to prevent the growth of immune-escaped transformed cells. Consistent with this, tumor-targeted silencing of TAP in mouse models resulted in potent antitumor immunity by inducing a common set of new antigens that are processed independently of TAP [102]. Therefore, TEIPP-specific T lymphocytes are promising effectors for the treatment of tumors that have evaded CD8 T-cell recognition and destruction. TEIPP are derived from ubiquitous non-mutant self-proteins that are not naturally loaded into MHC-I molecules of healthy cells because they express normal levels of TAP. They constitute a hidden class of immunogenic non-mutant shared neoantigens that appear on the cancer cell membrane upon tumor immune evasion and represent promising candidates for targeted therapeutic cancer vaccination [99, 100].

## Conclusions

Recent technological advances in identifying mutation-derived tumor antigens have enabled the development of patient-specific therapeutic vaccines that target individual cancer mutations. The association of mutant neoantigens with shared TAA and non-mutant neoepitopes would allow targeting tumor heterogeneity to eliminate all types of malignant cells, including those with APM defects. These polyepitope cancer vaccines combined with ICB will broaden T-cell specificities and reinvigorate exhausted antitumor CTL. However, a difficulty remains in the selection of the type of antigens to include in the vaccine. The conformation of the antigen and the targeted HLA could promote distinct responses between individual patients [76, 103]. Therefore, a vaccine designed with specific antigens targeting multiple HLA molecules could result in better efficacy. In this context, personalized cancer vaccines offer promise with the opportunity to treat tumors that have acquired resistance to ICB immunotherapies. However, a critical aspect is the long process needed to identify neoantigens for each individual patient and the cost of the technology. Another remaining challenge for neoantigen-based vaccination is to overcome T-cell exhaustion and immunosuppression. As future prospects, the evaluation of cancer vaccines in combination with ICB or other forms of immunotherapy needs to be intensified to improve the benefits for patients.

## Abbreviations

APC: antigen-presenting cell
APM: antigen presentation machinery
CTLs: cytotoxic T lymphocytes
DCs: dendritic cells
HER2: human epidermal growth factor receptor 2
HLA-I: human leukocyte antigen class I
HPV: human papillomavirus
ICB: immune checkpoint blockade
IL-2: interleukin 2
MAGE-1: melanoma-associated antigen-1
MHC-I: major histocompatibility complex class I
mRNA: messenger RNA
MUC1: mucin 1
NSCLC: non-small cell lung cancer
NY-ESO-1: New York esophageal squamous cell carcinoma 1
PD-1: programmed death-1
ppCT: preprocalcitonin
PRAME: preferentially expressed antigen in melanoma
RNAseq: RNA sequencing
TAAs: tumor-associated antigens
TAP: transporter associated with antigen processing
TCR: T-cell receptor
TEIPP: T-cell epitopes associated with impaired peptide processing
WES: whole exome sequencing
β_2_m: beta-2-microglobulin

## Declarations

### Copyright

© The Author(s) 2022.
